# Does using artificial intelligence assistance accelerate skill decay and hinder skill development without performers’ awareness?

**DOI:** 10.1186/s41235-024-00572-8

**Published:** 2024-07-12

**Authors:** Brooke N. Macnamara, Ibrahim Berber, M. Cenk Çavuşoğlu, Elizabeth A. Krupinski, Naren Nallapareddy, Noelle E. Nelson, Philip J. Smith, Amy L. Wilson-Delfosse, Soumya Ray

**Affiliations:** 1 Case Western Reserve University, 10900 Euclid Ave., Cleveland, OH 44106 USA; 2 Emory University, 1364 Clifton Road NE, Atlanta, GA 30322 USA; 3 The Ohio State University, 1971 Neil Ave., Columbus, OH 43210 USA

**Keywords:** Artificial intelligence, Expertise, Skill acquisition, Skill decay

## Abstract

Artificial intelligence in the workplace is becoming increasingly common. These tools are sometimes used to aid users in performing their task, for example, when an artificial intelligence tool assists a radiologist in their search for abnormalities in radiographic images. The use of artificial intelligence brings a wealth of benefits, such as increasing the efficiency and efficacy of performance. However, little research has been conducted to determine how the use of artificial intelligence assistants might affect the user’s cognitive skills. In this theoretical perspective, we discuss how artificial intelligence assistants might accelerate skill decay among experts and hinder skill acquisition among learners. Further, we discuss how AI assistants might also prevent experts and learners from recognizing these deleterious effects. We then discuss the types of questions: use-inspired basic cognitive researchers, applied researchers, and computer science researchers should seek to answer. We conclude that multidisciplinary research from use-inspired basic cognitive research, domain-specific applied research, and technical research (e.g., human factors research, computer science research) is needed to (a) understand these potential consequences, (b) design artificial intelligence systems to mitigate these impacts, and (c) develop training and use protocols to prevent negative impacts on users’ cognitive skills. Only by answering these questions from multidisciplinary perspectives can we harness the benefits of artificial intelligence in the workplace while preventing negative impacts on users’ cognitive skills.

## Introduction

Consider a radiologist examining a medical image for abnormalities. It is increasingly likely they will be aided by an artificial intelligence (AI) assistant (Hosny et al., 2018). The AI assistant may be a machine learning-based classifier that has been trained on a large number of images to, for example, classify abnormalities in breast images as benign or malignant. Given a new image, the AI highlights regions with presumed abnormalities or provides heatmaps, probabilities, graphs, or written descriptions. Such decision support systems are increasingly common in medicine and other areas (Magrabi et al., 2019).

As another example, the use of robots in surgical operations began over thirty years ago (see Gomes, 2011). Computer scientists are working to develop AI tools to assist the surgeon during operations. In the future, these AI assistants might gently nudge the surgeon to guide the surgical tools to the proper location, suggest the appropriate surgical tool for the task, and complete subcomponents of the procedure (Panesar et al., 2019).

In this theoretical perspective, we discuss the possibility that relying on AI assistants, including those yet to be developed, may have detrimental effects on human cognitive skills, such as expert judgment and decision making, integration of declarative knowledge, and problem solving. We use two subdisciplines within the field of medicine as examples: radiology, where artificial intelligence is currently being used in clinical practice, and robotic surgery, where artificial intelligence assistants are being developed to assist experts in the future. We first briefly describe cognitive processes involved in expertise and skill development. We then describe how the use of automated systems affects people’s cognitive skills and why consistent use of AI assistants, which differ from other forms of automation, may be detrimental to human skill learning and expertise. Specifically, we describe potential AI-induced skill decay and potential AI-induced skill development hindrance. Finally, we propose future directions that harness research from multiple perspectives are needed to understand these problems and develop solutions.

## Learning and expertise

We acquire many skills over our lifetime to varying degrees of proficiency. Often, people initially rely on inefficient algorithms that are high in cognitive effort, such as children counting on their fingers to solve basic addition problems (Bajic & Rickard, 2011). With practice, we improve our performance, and for many skills, the amount of cognitive effort also dramatically decreases with practice (Fitts & Posner, 1967). Inefficient algorithms are replaced with more efficient algorithms or direct retrieval, such as adults directly accessing the answer to “2 + 2” from memory rather than walking through steps of a calculation (Ackerman & Woltz, 1994; Logan, 1988).

Depending on the domain, practice designed to improve domain-specific performance tends to explain between 0 and 20% of variance in performance (Macnamara et al., 2014). For simple tasks that tap automated processes (Schneider & Shiffrin, 1977; Shiffrin & Schneider, 1977), skill development emerges quickly with practice before asymptoting (Fitts & Posner, 1967). Individual differences in cognitive abilities are relevant initially, but less so after accumulating practice (Ackerman, 1986). In contrast, for complex tasks that tap controlled processes (as opposed to automated processes; Schneider & Shiffrin, 1977; Shiffrin & Schneider, 1977), skill development emerges more slowly and is highly dependent on individual differences (e.g., Ackerman, 1986). In tasks that demand controlled processes, practice has limited predictive power because people vary in their starting points, learning rates, and apogees, such that an hour of practice for one person does not confer the same benefit as an hour of practice for someone else (Macnamara et al., 2023).

Because many complex tasks continue to rely on cognitive resources (Ackerman, 1986), individual differences in cognitive abilities often predict skill acquisition rates and levels of ultimate expertise. For example, working memory capacity predicts music sight-reading performance (Meinz & Hambrick, 2010) and Texas Hold’em expertise (Meinz et al., 2012); fluid intelligence predicts piano skill acquisition (Burgoyne et al., 2019) and reading competence (Lechner et al., 2019); and cognitive processing speed predicts air traffic control skill levels (Ackerman, 1988) and simultaneous language interpreting expertise (Macnamara et al., 2011). Further, the predictive strength of both experiential and cognitive factors depends on characteristics of the task, such as the consistency of stimuli-response mappings (Ackerman, 1986; Macnamara & Frank, 2018; Macnamara et al., 2014), level of concurrence among subtasks (Frank & Macnamara, 2021; Hoffman et al., 2014), and rate of stimuli change (Hoffman et al., 2014; Macnamara & Frank, 2018; Macnamara et al., 2014). Given the demands of complex tasks on people’s cognitive resources, technological advances have been made to reduce cognitive load and human error during complex task performance.

### The use of automation and artificial intelligence

Automation has been used for decades across multiple industries. Perhaps the most studied use of automation on human mental processes is the autopilot in aviation. Autopilots are designed to perform some of the human pilot’s tasks, such as landing the plane and keeping the wings level during the flight. Without autopilot, flying demands continuous attention from the human pilot. For longer flights, mental fatigue from the continuous attention became a safety threat. Thus, the use of automation has improved safety and flight performance (Federal Aviation Administration, 2013).

Despite these benefits, automation can also lead to bias and complacency, resulting in worse flight performance. When an automated system provides information to a user, this information can bias the user’s situation assessment (Smith et al., 1997), change their visual scanning patterns (Rezazade Mehrizi et al., 2023), and reduce how frequently they cross-check information (Manzey et al., 2012). In some cases, users are prone to favor information from automated systems over human expertise, even when the recommendations conflict and the automated suggestions are inaccurate (automation bias; Manzey et al., 2012; Mosier et al., 1997).

Other research indicates that using automation can lead to reliance on the automated system. Reliance can positively affect performance, such as when users appropriately trust the system to handle one part of the workload while the user directs additional attention to other components of the task. In contrast, reliance can negatively affect performance when users fail to evaluate the performance of automated systems and allow system errors, malfunctions, or anomalous conditions to go undetected (automation-induced complacency; Risko & Gilbert, 2016). Whether reliance is beneficial or detrimental depends on the accuracy of the automated system and how much trust the user has in the system. Quantitative syntheses of the literature suggest that the ‘crossover point’ from benefits to detriments, at least in high workload conditions, is around 70% accuracy (Parasuraman & Manzey, 2010; Wickens & Dixon, 2007). Yet, some users continued to rely on systems with low accuracy, such that their performance would have been better with no automation at all (Wickens & Dixon, 2007).

Automation bias and automation-induced complacency are not the same concerns as automation affecting a person’s skill. In other words, if the automation was removed, the user would no longer exhibit bias or automation-induced complacency. But what about the person’s skill? Some research has focused on how technological assistance might cause cognitive skill decay (Ebbatson et al., 2010; Kim et al., 2013; Kluge & Frank, 2014; Smith & Baumann, 2019; Volz et al., 2016), particularly in aviation (e.g., Casner et al., 2014; Ebbatson et al., 2010; Haslbeck & Hoerman, 2016). As an example, these studies raise a concern that the more time spent flying with automation support, the worse pilots’ performance was when this automation was unavailable or irrelevant (e.g., handling an aviation emergency, hand flying an approach). After a rise in near misses and other evidence that pilot’s manual skills were declining (Leahy & Fisher, 2023), the U.S. Federal Aviation Administration has recommended that pilots periodically use their manual skills for the majority of flights (Federal Aviation Administration, 2022).

Notably, automated systems, such as most autopilot systems, differ from artificial intelligence assistants. Automation refers to systems where the same input would repeatedly produce the same results. Automated system performance is governed by rules explicitly coded by developers. For example, airplane autopilots are automated control systems with a decision/feedback control loop based on known electromechanical characteristics of the airplane within normal parameters. When outside of normal parameters, such automation support will typically disengage and hand control to the pilots—they do not actively assist with expert decision making to the extent of AI.

In contrast, AI technologies are designed to mimic expert decision making by dynamically assessing problems, providing adaptive and novel solutions, and performing tasks that are cognitively demanding for humans (Walsh et al., 2019). These technologies come in multiple forms, from natural language processing to decision support systems (Bankins et al., 2023; Walsh et al., 2019). Here, we focus on a class of AI technologies—human-AI collaboration—often discussed in the future of work designed to aid humans in their jobs or skill development (Bankins et al., 2023; Brynjolfsson, 2023). More specifically, we refer to AI assistants as AI technologies (both current and future) that aid humans in problem solving, performance in cognitively demanding tasks, and in skill development. Examples include AI tools designed to aid radiologists during image analysis and AI tools designed to aid surgeons in their decisions and performance during robotic surgery operations. Current and future AI assistants go beyond automation to aid in high-level decision making that was previously the expert’s domain alone. To our awareness, no research has been conducted on AI assistants’ effects on the user’s skill.

### Potential AI-induced skill decay

Research on skill decay suggests that after a period of disuse, skilled performance declines (Ackerman & Tatel, 2023; Arthur et al., 2013). A factor that influences this decay is the extent of cognitive demand in the task. More specifically, higher levels of skill decay are associated with tasks that require greater cognitive effort and fewer physical demands (i.e., controlled processes), as opposed to tasks that primarily rely on physical abilities with minimal cognitive processing (i.e., proceduralized skills) (Arthur et al., 1998; Wang et al., 2013).^1^

In addition to ceasing the task altogether, disuse can also refer to ceasing subcomponents of a task, such as when automation is used in place of the person applying their skills. As an illustration, Casner et al. (2014) tested the manual flying skills of pilots who were trained to fly manually, but then spent the majority of their careers flying with high automation (i.e., autopilot for much of takeoff, cruising, and landing). Procedural skills, such as scanning the instruments and manual control, were “rusty” but largely intact. In contrast, major declines in cognitive skills emerged, such as failures to maintain awareness of the airplane’s location, keep track of next steps, make planned changes along the route, and recognize and handle instrument systems failures.

To a much higher degree than automated systems, artificial intelligence assistants are designed to mimic cognitive skills—that is, they recognize patterns, reason about potential outcomes, and often guide the user to a specific action. For this reason, consistent and repeated engagement with an AI assistant is likely to lead to greater decrements in skill than engagement with automation systems. Frequent engagement with an AI assistant designed to take over some of the user’s cognitive processes means there are fewer opportunities to keep skills honed.

The potential for AI-induced skill decay is particularly relevant in high-risk industries, medical fields, aviation, and military operations, where maintaining high-level skills is essential for performance and safety (Day et al., 2013; Villado et al., 2013). Though some fields will flourish as AI improves in accuracy and takes over tasks, thereby reducing human error, other fields will have problems that AI cannot solve. For example, in medicine, new problems, such as a novel virus with unforeseen symptoms, can emerge at any time. Given that AI needs to be trained with data, human experts with keen insights are needed to solve novel problems. Such fields will benefit by maintaining human experts’ skills.

A potential complicating factor is that experts may be unaware of their skill decay. In cases where a person has ceased the task altogether, they will be aware that they have not used their skills and may be more likely to be aware of their skill decay. In contrast, AI-induced skill decay may operate outside the performer’s awareness because the disuse is only at the level of cognitive skill engagement, not with engagement with the task. As such, a surgeon using an AI assistant may believe their skills are still sharp because they have continued to perform operations at a high level (i.e., successful surgeries). They may not consider how well they would be able to perform without the AI assistant, such as selecting appropriate operative techniques, navigating complex anatomy (e.g., by identifying relevant anatomical landmarks and dissecting tissue to access target anatomy), or optimizing visualization (through changing laparoscopic camera angles and/or through dissection and retraction of tissue). Likewise, a radiologist using an AI assistant may believe they are highly capable of detecting and classifying anomalies in images, but their skills needed to discriminate between similar-appearing abnormalities, grade abnormalities severity properly, assess tissue density, measure tumor dimensions accurately, or detect subtle abnormalities may be less sharp than they believe. Both AI-induced skill decay and experts’ awareness of their skill decay are critical questions for use-inspired basic research, technical research, and applied research.

### Potential AI-induced skill development hindrance

As AI assistants become increasingly prevalent, the role AI might have on skill development needs to be considered. AI-learning aids are designed to improve the rate of learner’s skill or knowledge development. We propose that frequent engagement with an AI assistant during skill development might hinder learning in some cases, depending on the ultimate goal for which the AI-learning aid was developed. Educational aids that are designed to personalize instruction for students with the goal of students independently performing the task are unlikely to hinder learning. In contrast, AI-learning aids designed to prepare trainees for work where AI assistants are used may focus on preparing the learner to work *with* an AI rather than focusing on developing learners’ cognitive skills *independent of* AI. For example, a radiologist trainee may not develop as keen visual detection skills or a surgical resident may not develop as robust spatial navigation skills as if they would have developed had they trained without assistance.

Here, it is important to distinguish between learning and performance. Suppose learners are randomly assigned to either learn a task with a high-performing AI assistant or without. We would expect the learners with the AI assistant to improve their performance rapidly and outperform the learners without AI. However, these performance gains may not reflect the learners’ gains in skill independent of the AI. That is, now suppose that after a period of learning that the AI is withheld, as may happen in the real world if the system is unavailable or fails. We might expect that the group who previously had access to the AI assistant to perform worse than those who never learned with the AI. In this case, those who learned with AI assistance might not have developed independent cognitive skills that the control group developed. Stated differently, we might expect to observe a pattern opposite of latent learning—high performance is observed in the AI-assisted group, but the limits on learning remain hidden until AI assistance is removed.

As with potential AI-induced skill decay, learners who have engaged with AI to assist in their skill development might be unaware of where their skills are lacking. In particular, AI assistants may promote illusions of understanding in learners, leading them to believe they have a greater understanding of the task than they actually do (Messeri & Crockett, 2024). These illusions of understanding may occur when learners believe they have a deeper understanding than they actually do (i.e., illusion of explanatory depth); when learners believe they are considering all possibilities rather than only those available through the AI assistant (i.e., illusion of exploratory breadth); and when learners believe that the AI assistant is objective, failing to consider the bias embedded in the AI tool from the developers and training data (i.e., illusion of objectivity) (Messeri & Crockett, 2024). As such, AI-induced skill development hindrance may not only limit the level of learning obtained but may change the nature of the understanding of the task.

## Conclusions, recommendations, and future directions

AI tools are unquestionably beneficial in many respects (e.g., improved diagnostic accuracy, fewer human errors). However, a concern is that there may be downsides for the people using them. In particular, if the AI routinely aids performance at a high level, even well-trained experts may gradually lose their task-based cognitive skills, instead relying on the AI to render decisions. Additionally, it may be that trainees do not build their own cognitive skills at the task effectively when the AI serves as a learning aid. Further, because AI tools are likely to enhance performance and make the task feel easier, learners may be less able to judge the true status of their skills and experts may be unaware of their deteriorating skills, resulting in performance degradation when relying on the AI is not optimal.

Though the degradation of human skills may be of little concern in domains where reliance on AI can approach optimal performance, medicine will likely continue to need human experts. AI will have difficulty handling new viruses, unique injuries from accidents, or individual context and significant variations in a patient’s physiology. Thus, as AI assistants become increasingly common, their effects on human skills need to be considered, especially in life-altering situations such as radiology or robotic surgery.

The available evidence suggests that frequent engagement with automation induces skill decay. Given that (a) AI is often designed to take over more advanced cognitive processes than non-AI automation, and (b) skill decay is accelerated for cognitive skills, AI-induced skill decay is a likely consequence of frequent engagement with AI assistants.

Depending on the field, AI-induced skill decay may only be a concern in the short-term. If AI assistants can be developed such that they are near perfect in all (or nearly all) circumstances in the future, then developing and maintaining human skills for that task may be unnecessary. But, in fields where problems rapidly evolve or novel events are likely to occur, such as in medicine, or if the AI tool is biased, offline, or making errors, then developing and maintaining human skills will continue to be advantageous.

**Fig. 1:**
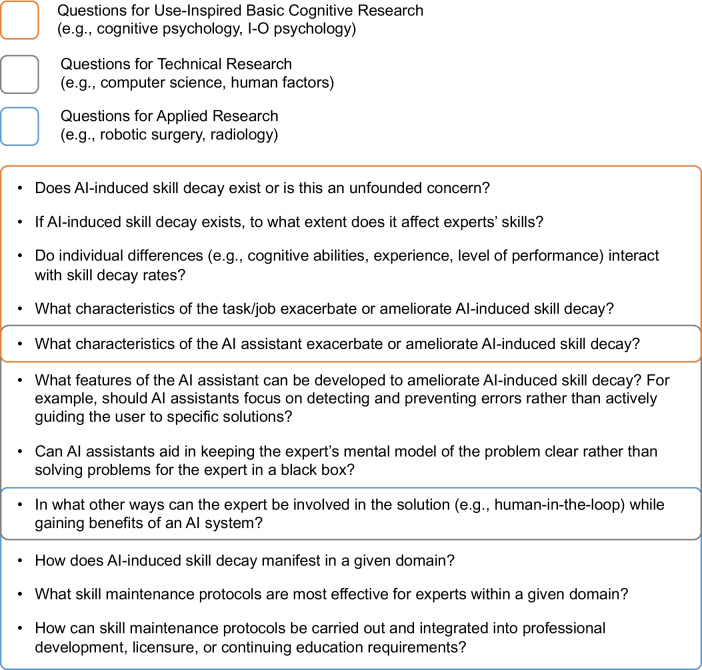
Suggested questions for research on AI-induced skill decay

**Fig. 2:**
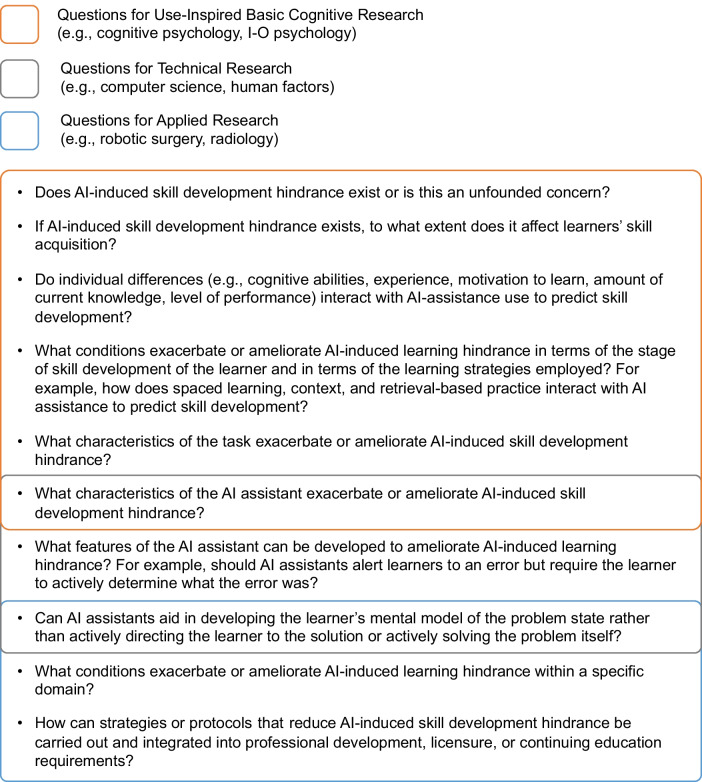
Suggested questions for research on AI-induced skill development hindrance

We propose several lines of inquiry (Figs. 1 and 2) that researchers from multiple perspectives should seek to address related to potential AI-induced skill decay and AI-induced skill development hindrance. The proposed research perspectives are not intended to be discrete areas or mutually exclusive. Likewise, the research questions we propose are not intended to be exhaustive or necessarily the most urgent or important but offer an initial sample of potential research lines in these areas.

Further, when addressing these questions, researchers should consider the degree of awareness experts and learners may (or may not) have on their level of skill. If AI assistants lead learners toward an illusion of understanding of the task (Messeri & Crockett, 2024) or lead experts to believe their skills are at a higher level than they are in reality, then basic research, technical research, and applied research are needed to understand the sources, moderators, and solutions to these issues. Only by drawing on multiple disciplinary perspectives can we best understand the impacts of artificial intelligence on humans’ cognitive skills and the future of workplace expertise.

## Open practices statement

This paper discusses a perspective on an emerging potential problem and recommends courses of action. We do not report on any studies or original data. Thus, the availability of data and/or materials is not applicable. Likewise, no experiments were conducted and, therefore, preregistration was not applicable.

## Data Availability

Not applicable.

## Footnotes

1 Skill decay of cognitive-oriented tasks may also exhibit varying decay patterns depending on their complexity (see, e.g., Villado et al., 2013).

## References

[CR1] Ackerman PL (1986). Individual differences in information processing: An investigation of intellectual abilities and task performance during practice. Intelligence.

[CR2] Ackerman PL (1988). Determinants of individual differences during skill acquisition: Cognitive abilities and information processing. Journal of Experimental Psychology: General.

[CR3] Ackerman PL, Tatel CE (2023). Resolving problems with the skill retention literature: An empirical demonstration and recommendations for researchers. Journal of Experimental Psychology: Applied.

[CR4] Ackerman PL, Woltz DJ (1994). Determinants of learning and performance in an associative memory/substitution task: Task constraints, individual differences, volition, and motivation. Journal of Educational Psychology.

[CR5] Arthur W, Bennett W, Stanush PL, McNelly TL (1998). Factors that influence skill decay and retention: A quantitative review and analysis. Human Performance.

[CR6] Arthur W, Day EA, Bennett W, Portrey AM (2013). Individual and team skill decay: The science and implications for practice.

[CR7] Bajic D, Rickard TC (2011). Toward a generalized theory of the shift to retrieval in cognitive skill learning. Memory & Cognition.

[CR8] Bankins S, Ocampo AC, Marrone M, Restubog SLD, Woo SE (2023). A multilevel review of artificial intelligence in organizations: Implications for organizational behavior research and practice. Journal of Organizational Behavior.

[CR9] Barker R (2011). 2030—The future of medicine: Avoiding a medical meltdown.

[CR10] Baron JM, Huang R, McEvoy D, Dighe AS (2021). Use of machine learning to predict clinical decision support compliance, reduce alert burden, and evaluate duplicate laboratory test ordering alerts. JAMIA Open.

[CR11] Brynjolfsson, E. (2023). A call to augment—not automate—workers. In *Generative AI: Perspectives from Stanford HAI*. Stanford University Human-Centered Artificial Intelligence. Retrieved March 24, 2024, from https://hai.stanford.edu/sites/default/files/2023-03/Generative_AI_HAI_Perspectives.pdf

[CR12] Burgoyne AP, Harris LJ, Hambrick DZ (2019). Predicting piano skill acquisition in beginners: The role of general intelligence, music aptitude, and mindset. Intelligence.

[CR13] Cohen SA (2009). A review of demographic and infrastructural factors and potential solutions to the physician and nursing shortage predicted to impact the growing US elderly population. Journal of Public Health Management and Practice.

[CR14] Casner SM, Geven RW, Recker MP, Schooler JW (2014). The retention of manual flying skills in the automated cockpit. Human Factors.

[CR15] Coratti A, Annecchiarico M (2013). Robotics in general surgery: Current status and critical review. OA Robotic Surgery.

[CR16] Dahmani L, Bohbot VD (2020). Habitual use of GPS negatively impacts spatial memory during self-guided navigation. Scientific Reports.

[CR17] Day EA, Arthur W, Villado AJ, Boatman PR, Kowollik V, Bhupatkar A, Bennett W, Arthur W, Day EA, Bennett W, Portray A (2013). Relating individual differences in ability, personality, and motivation to the retention and transfer of skill on a complex command-and-control simulation task. Individual and team skill decay.

[CR18] Ebbatson M, Harris D, Huddlestone J, Sears R (2010). The relationship between manual handling performance and recent flying experience in air transport pilots. Ergonomics.

[CR19] Federal Aviation Administration. (2013). Safety alert for operators: Manual flight operations. SAFO 13002. Retrieved March 24, 2024, from https://www.faa.gov/sites/faa.gov/files/other_visit/aviation_industry/airline_operators/airline_safety/SAFO13002.pdf

[CR20] Federal Aviation Administration. (2022). Advisory circular: Flightpath management. Advisory circular number 120–123. Retrieved February 29, 2024, from https://www.faa.gov/documentLibrary/media/Advisory_Circular/AC_120-123.pdf

[CR21] Fiorini P, Goldberg KY, Liu Y, Taylor RH (2022). Concepts and trends in autonomy for robot-assisted surgery. Proceedings of the IEEE.

[CR22] Fitts PM, Posner MI (1967). Human performance.

[CR23] Frank DJ, Demaree HA, Macnamara BN (2020). The role of individual differences in risk learning: Who learns to place optimal wagers?. Learning and Motivation.

[CR24] Frank DJ, Macnamara BN (2021). How do task characteristics affect learning and performance? The roles of simultaneous, interactive, and continuous tasks. Psychological Research Psychologische Forschung.

[CR25] Ghaith GM (2002). The relationship between cooperative learning, perception of social support, and academic achievement. System.

[CR26] Gomes P (2011). Surgical robotics: Reviewing the past, analysing the present, imagining the future. Robotics and Computer-Integrated Manufacturing.

[CR27] Goodman JS, Wood RE (2004). Feedback specificity, learning opportunities, and learning. Journal of Applied Psychology.

[CR28] Grier DA (2005). When computers were human.

[CR29] Haslbeck A, Hoerman H-J (2016). Flying the needles: Flight deck automation erodes fine-motor flying skills among airline pilots. Human Factors.

[CR30] Hoffman RR, Ward P, Feltovich PJ, DiBello L, Fiore SM, Andrews DH (2014). Accelerated expertise: Training for high proficiency in a complex world.

[CR31] Hosny A, Parmer C, Quuackenbush J, Schwartz LH, Aerts HJWL (2018). Artificial intelligence in radiology. Nature Reviews Cancer.

[CR32] Kim JW, Ritter FE, Koubek RJ (2013). An integrated theory for improved skill acquisition and retention in the three stages of learning. Theoretical Issues in Ergonomics Science.

[CR33] Kluge A, Frank B (2014). Counteracting skill decay: Four refresher interventions and their effect on skill and knowledge retention in a simulated process control task. Ergonomics.

[CR34] Krupinski, E. A. (2000). Medical image perception: Evaluating the role of experience. In *Human Vision and Electronic Imaging V*, *Proceedings Volume 3959*, 281–289). 10.1117/12.387164

[CR35] Leahy, J., & Fisher, A. (2023). FAA shifts focus to pilot manual handling skills. *Royal Aeronautical Society*. Retrieved February 29, 2024, from https://www.aerosociety.com/news/faa-shifts-focus-to-pilot-manual-handling-skills/

[CR36] Lechner CM, Miyamoto A, Knopf T (2019). Should students be smart, curious, or both? Fluid intelligence, openness, and interest co-shape the acquisition of reading and math competence. Intelligence.

[CR37] Logan GD (1988). Toward an instance theory of automatization. Psychological Review.

[CR38] Macnamara BN, Frank DJ (2018). How do task characteristics affect learning and performance? The roles of variably mapped and dynamic tasks. Journal of Experimental Psychology: Learning, Memory, and Cognition.

[CR39] Macnamara BN, Hambrick DZ, Oswald FL (2014). Deliberate practice and performance in music, games, sports, education, and professions: A meta-analysis. Psychological Science.

[CR40] Macnamara BN, Moore AB, Kegl JA, Conway AR (2011). Domain-general cognitive abilities and simultaneous interpreting skill. Interpreting.

[CR41] Macnamara BN, Prather RW, Burgoyne AP (2023). Beliefs about success are prone to cognitive fallacies. Nature Reviews Psychology.

[CR42] Magrabi F, Ammenwerth E, McNair JB, De Keizer NF, Hyppönen H, Nykänen P, Rigby M, Scott PJ, Vehko T, Wong ZS-Y, Georgiou A (2019). Artificial intelligence in clinical decision support: Challenges for evaluating AI and practical implications. Yearbook of Medical Informatics.

[CR43] Manzey D, Reichenbach J, Onnasch L (2012). Human performance consequences of automated decision aids: The impact of degree of automation and system experience. Journal of Cognitive Engineering and Decision Making.

[CR44] Meinz EJ, Hambrick DZ (2010). Deliberate practice is necessary but not sufficient to explain individual differences in piano sight-reading skill: The role of working memory capacity. Psychological Science.

[CR45] Meinz EJ, Hambrick DZ, Hawkins CB, Gillings AK, Meyer BE, Schneider JL (2012). Roles of domain knowledge and working memory capacity in components of skill in Texas Hold’Em poker. Journal of Applied Research in Memory and Cognition.

[CR46] Messeri L, Crockett MJ (2024). Artificial intelligence and illusions of understanding in scientific research. Nature.

[CR47] Mosier KL, Skitka LJ, Heers S, Burdick M (1997). Automation bias: Decision making and performance in high-tech cockpits. International Journal of Aviation Psychology.

[CR48] Nestojko JF, Finley JR, Roediger HL (2014). Extending cognition to external agents. Psychological Inquiry.

[CR49] Panesar S, Cagle Y, Chander D, Morey J, Fernandez-Miranda J, Kliot M (2019). Artificial intelligence and the future of surgical robotics. Annals of Surgery.

[CR50] Parasuraman R, Manzey DH (2010). Complacency and bias in human use of automation: An attentional integration. Human Factors.

[CR51] Rezazade Mehrizi MH, Mol F, Peter M, Ranschaert E, Dos Santos DP, Shahidi R, Fatehi M, Dratsch T (2023). The impact of AI suggestions on radiologists’ decisions: A pilot study of explainability and attitudinal priming interventions in mammography examination. Nature Scientific Reports.

[CR52] Risko EF, Gilbert SJ (2016). Cognitive offloading. Trends in Cognitive Sciences.

[CR53] Roediger HL, Karpicke JD (2006). Test-enhanced learning: Taking memory tests improves long-term retention. Psychological Science.

[CR54] Schneider W, Shiffrin RM (1977). Controlled and automatic human information processing: I. Detection, search, and attention. Psychological Review.

[CR55] Shiffrin RM, Schneider W (1977). Controlled and automatic human information processing: II. Perceptual learning, automatic attending and a general theory. Psychological Review.

[CR56] Smith, P. J., & Baumann, E. (2019). Human-automation teaming: Unintended impacts and mitigations for degraded NextGen operations. https://rosap.ntl.bts.gov/view/dot/43777

[CR57] Smith PJ, McCoy E, Layton C (1997). Brittleness in the design of cooperative problem-solving systems: The effects on user performance. IEEE Transactions on Systems, Man and Cybernetics.

[CR58] Smith SM, Glenberg A, Bjork RA (1978). Environmental context and human memory. Memory & Cognition.

[CR59] Villado AJ, Day EA, Arthur W, Boatman PR, Kowollik V, Bhupatkar A, Bennett W, Arthur W, Day EA, Bennett W, Portray A (2013). Complex command-and-control simulation task performance following periods of nonuse. Individual and team skill decay.

[CR60] Volz K, Yang E, Dudley R, Lynch E, Dropps M, Dorneich M (2016). An evaluation of cognitive skill degradation in information automation. Proceedings of the Human Factors and Ergonomics Society Annual Meeting.

[CR61] Walsh, T., Levy, N., Bell, G., Elliott, A., Maclaurin, J., Mareels, I., & Wood, F. M. (2019). *The effective and ethical development of artificial intelligence: An opportunity to improve our wellbeing*. Australian Council of Learned Academies. Retrieved March 24, 2024, from https://acola.org/wp-content/uploads/2019/07/hs4_artificial-intelligence-report.pdf

[CR62] Wang, X., Day, E. A., Kowollik, V., Schuelke, M. J., & Hughes, M. G. (2013). Factors influencing knowledge and skill decay after training: A meta-analysis. In *Individual and Team Skill Decay* (pp. 68–116). Routledge.

[CR63] Wickens CD, Dixon SR (2007). The benefits of imperfect diagnostic automation: A synthesis of the literature. Theoretical Issues in Ergonomics Science.

[CR64] Yang GZ, Cambias J, Cleary K, Daimler E, Drake J, Dupont PE, Hata N, Kazanzides P, Martel S, Patel RV, Santos VJ, Taylor RH (2017). Medical robotics—Regulatory, ethical, and legal considerations for increasing levels of autonomy. Science Robotics.

